# Pain Persistence Is Associated With Increased Odds of MCI in Late Midlife and Early Older Adulthood

**DOI:** 10.1093/geroni/igab046.438

**Published:** 2021-12-17

**Authors:** Tyler Bell, Jeremy Elman, Carol Franz, William Kremen

**Affiliations:** University of California San Diego, La Jolla, California, United States

## Abstract

Twenty percent of older adults will experience persistent pain, the sensation of bodily harm lasting three or more months. Persistent pain doubles the risk of dementia, but we know less about the impact on earlier stages, such as mild cognitive impairment (MCI). As a step for clarification, this study leveraged data from the Vietnam Era Twin Study of Aging (VETSA) to understand how pain persistence relates to MCI in late midlife to early older adulthood. Participants (n=1,465, 100% male) were recruited across three waves at average ages 56, 62, and 68. At each wave, participants completed the SF-36 and were asked to rate their pain intensity from none (1) to very severe (6). Clinical pain was coded as pain intensity rated more than mild (>3/6). As a time-varying predictor, pain persistence was then calculated as a running frequency of the total waves reporting clinical pain. MCI diagnosis was based on Jak-Bondi criteria. Age, depressive symptoms, comorbidities, and opioid use were included as time-varying covariates. Age and education were included as time-invariant covariates. General estimating equations showed that pain persistence over two waves, reported in 35% of the sample, increased MCI odds by 57% (OR=1.57, 95%CI: 1.28 to 1.94). Pain persistence over three waves, reported in 17% of the sample, increased MCI odds by 98% (OR=1.98, 95%CI: 1.44 to 2.70). The findings emphasize the role of pain in earlier stages of dementia and the potential importance of pain management in offsetting cognitive decline.

